# Time-course single-cell RNA sequencing reveals transcriptional dynamics and heterogeneity of limbal stem cells derived from human pluripotent stem cells

**DOI:** 10.1186/s13578-021-00541-4

**Published:** 2021-01-23

**Authors:** Changbin Sun, Hailun Wang, Qiwang Ma, Chao Chen, Jianhui Yue, Bo Li, Xi Zhang

**Affiliations:** 1 BGI-Shenzhen, Shenzhen, 518083 China; 2 BGI Education Center, University of Chinese Academy of Sciences, Shenzhen, 518083 China; 3 China National GeneBank, BGI-Shenzhen, Shenzhen, 518082 China; 4 Department of Radiation Oncology, School of Medicine, Johns Hopkins University, Baltimore, MD 21218 USA; 5 Section of Cell Biology and Physiology, Department of Biology, University of Copenhagen, Copenhagen, Denmark

**Keywords:** LSCs, scRNA-seq, Identity, Developmental trajectory

## Abstract

**Background:**

Human pluripotent stem cell-derived limbal stem cells (hPSC-derived LSCs) provide a promising cell source for corneal transplants and ocular surface reconstruction. Although recent efforts in the identification of LSC markers have increased our understanding of the biology of LSCs, much more remains to be characterized in the developmental origin, cell fate determination, and identity of human LSCs. The lack of knowledge hindered the establishment of efficient differentiation protocols for generating hPSC-derived LSCs and held back their clinical application.

**Results:**

Here, we performed a time-course single-cell RNA-seq to investigate transcriptional heterogeneity and expression changes of LSCs derived from human embryonic stem cells (hESCs). Based on current protocol, expression heterogeneity of reported LSC markers were identified in subpopulations of differentiated cells. EMT has been shown to occur during differentiation process, which could possibly result in generation of untargeted cells. Pseudotime trajectory analysis revealed transcriptional changes and signatures of commitment of hESCs-derived LSCs and their progeny—the transit amplifying cells.

**Conclusion:**

Single-cell RNA-seq revealed time-course expression changes and significant transcriptional heterogeneity during hESC-derived LSC differentiation in vitro. Our results demonstrated candidate developmental trajectory and several new candidate markers for LSCs, which could facilitate elucidating the identity and developmental origin of human LSCs in vivo.

## Background

Human limbal stem cells (LSCs) are located at a narrow area around the cornea and connect directly to the sclera [1–3]. Other than self-renewal capability for homeostasis maintenance, LSCs have unipotency to differentiated into corneal epithelial cells and play vital roles in corneal regeneration and repair [4]. However, internal or external factors, such as genetic mutations, chemicals, burns, bacteria etc., could result in limbal malfunction, and limbal stem cells deficiency (LSCD), and lead to reduced vision and blindness [5–7].

Among different treatment options, LSC transplantation is currently the best curative treatment that can improve both vision and quality-of-life in patients with ocular surface disorders caused by LSCD [8]. But the potential risks of infection during LSCs harvesting from donors and immunological rejection after transplantation hinder its broad application in the clinic. One promising alternative is the use of non-immunogenic limbal stem cells induced from engineered pluripotent stem cells, such as embryonic stem cells (ESCs) and induced pluripotent stem cells (iPSCs) [9–13]. However, the complexity and heterogeneity of the LSC niche, and our limited knowledge about the development of human LSCs prevent us from developing reliable and efficient methods for LSCs differentiation [13, 14].

Although the developmental origin of LSC remains enigmatic, most studies considered that the corneal epithelium descend from surface ectoderm (SE) [14, 15], which also give rise to the epidermis and ectodermal associated appendages such as hair, ears, and the mammary glands etc. [16]. However, developmental surface ectodermal cells and their derivatives are difficult to isolate and study in human. Our understanding on cell-fate specification of the limbal stem cells in vivo are largely from studies of classic model organisms, such as mice [17, 18] and *Xenopus* frogs [19]. But it is well-known that final maturation pathways are significantly different between humans and other model animals, though their pre-implantation development appear relatively similar [20]. Thus, the directed differentiation of human pluripotent stem cells (hPSCs) to LSCs could offer an alternative model system to explore these cells’ identity and fate decisions for basic and clinical applications [9–12, 16, 21]. However, available differentiation protocols are still inefficient and suffer from excessive heterogeneity [22]. The lack of specific markers for LSCs, and our limited knowledge about intrinsic signaling cascades and developmental mechanisms of human LSCs hindered the clinical application of LSCs [14, 21].

Single-cell RNA sequencing (scRNA-seq) is a powerful tool to quantify transcripts in individual cells to understand gene expression changes at single-cell resolution [23]. Since the first publication in 2009 [24], scRNA-seq has been increasingly utilized in many fields, such as in developmental biology to delineate cell lineage relationships and developmental trajectories [25–27]. In this study, we performed a time-course single-cell transcriptomic analysis of LSCs derived from human embryonic stem cells to investigate their transcriptional heterogeneity and expression changes during differentiation process in vitro.

## Results

### Single-cell RNA sequencing revealed expression heterogeneity in hESC-derived LSCs

H9 human embryonic stem cells (hESC) were converted to LSCs via a surface ectodermal stage according to previous published protocols [15, 28] (Additional file 1: Fig. S1a). To characterize obtained hESC-derived LSCs, we performed scRNA-seq at four time points: Day 0 before induction, Day 7, Day 14, and Day 21 after induction. In total, 18 541 cells were sequenced, and data from 14 241 cells were used for the following analysis after filtering out low quality cells, including 4 687 cells, 4 784 cells, 3 210 cells, and 1 560 cells from Day 0, Day 7, Day 14, and Day 21, respectively (Additional file 1: Fig. S1b-S1e).

**Fig. 1:**
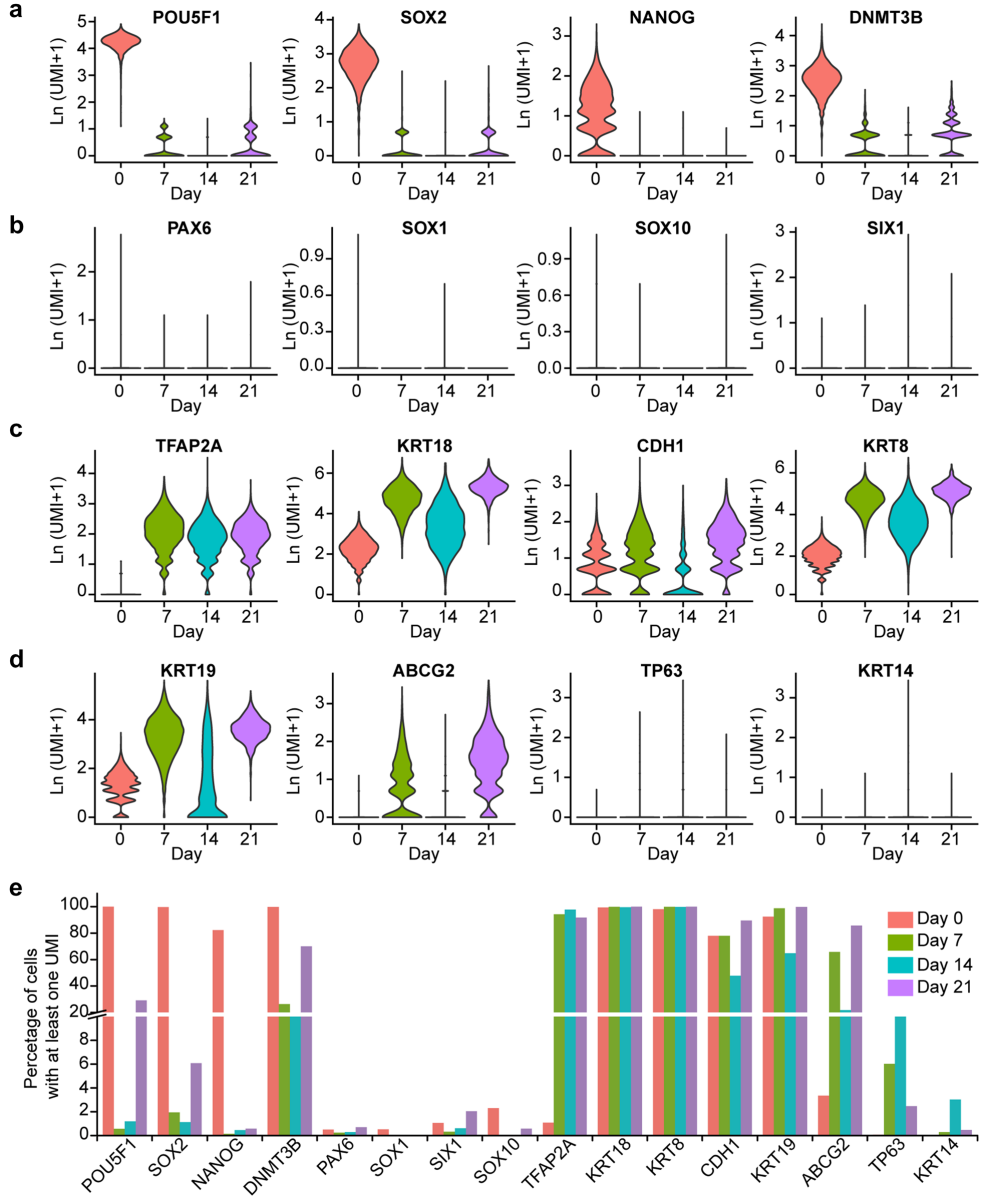
Single-cell RNA sequencing analysis of human embryonic stem cells-derived LSCs differentiation at days 0, 7, 14, and 21. **a-d** Violin plots representing expression (ln (UMI + 1)) of pluripotency (**a**), neural ectoderm (**b**), surface ectoderm and epithelium (**c**), and candidate LSCs (**d**) related markers at days 0, 7, 14, and 21. **e** Barplot representing percentage of cells expressed (at least with 1 UMI) of the selected pluripotency, neural ectoderm, surface ectoderm and epithelium, and candidate LSCs related markers at days 0, 7, 14, and 21

Gene expression analysis showed that, pluripotent markers, POU5F1, SOX2, and NANOG, were highly expressed in most cells at Day 0, accounting for 99.98%, 99.73%, and 82.27% of all the analyzed cells, respectively (Fig. 1a and e), which indicated that these cells used for hESC-derived LSCs differentiation were pluripotent.

At Day 7, 94.25% of cells expressed surface ectodermal marker TFAP2A while only a few of the cells expressed pluripotency markers (POU5F1 0.57%, SOX2 1.94%, NANOG 0.14%), neuroectodermal markers (SOX1 0.00%, PAX6 0.24%), neural crest marker (SOX10 0.04%), and cranial placode marker (SIX1 0.32%) (Fig. 1a, b and e), demonstrating almost no residual pluripotency and a direction of differentiation toward surface ectodermal progenitors [16]. In addition, a range of epithelial progenitor and candidate LSC markers [14], such as KRT19, KRT18, TP63, CDH1, and ABCG2, were expressed in this population (Fig. 1c–e). However, some of these genes, like TP63 (well-known as p63), which has been linked to successful limbal transplantation [29], only expressed in a small portion of cells (6.034%) (Fig. 1e).

We also found high variability in the expression of some epithelial progenitor and candidate LSCs markers between Day 14 and Day 21. Percentage of cells expressing TP63 decreased from 11.21% at Day 14 to 2.46% at Day 21 (Fig. 1e). In contrast, most cells (85.67%) at Day 21 expressed ABCG2, one of the widely used markers of LSCs [14, 30–32], while only 21.82% of cells at Day 14 had ABCG2 expression. Furthermore, several markers of terminally differentiated LSCs, such as KRT3 and KRT12 (data not shown), were not detected in any cells at Day 14 and Day 21, indicating that these cells were still at immature differentiation stages.

### Time-course Single-cell RNA-seq profiling showed specific changes of gene expression during hESCs-derived LSCs differentiation

**Fig. 2:**
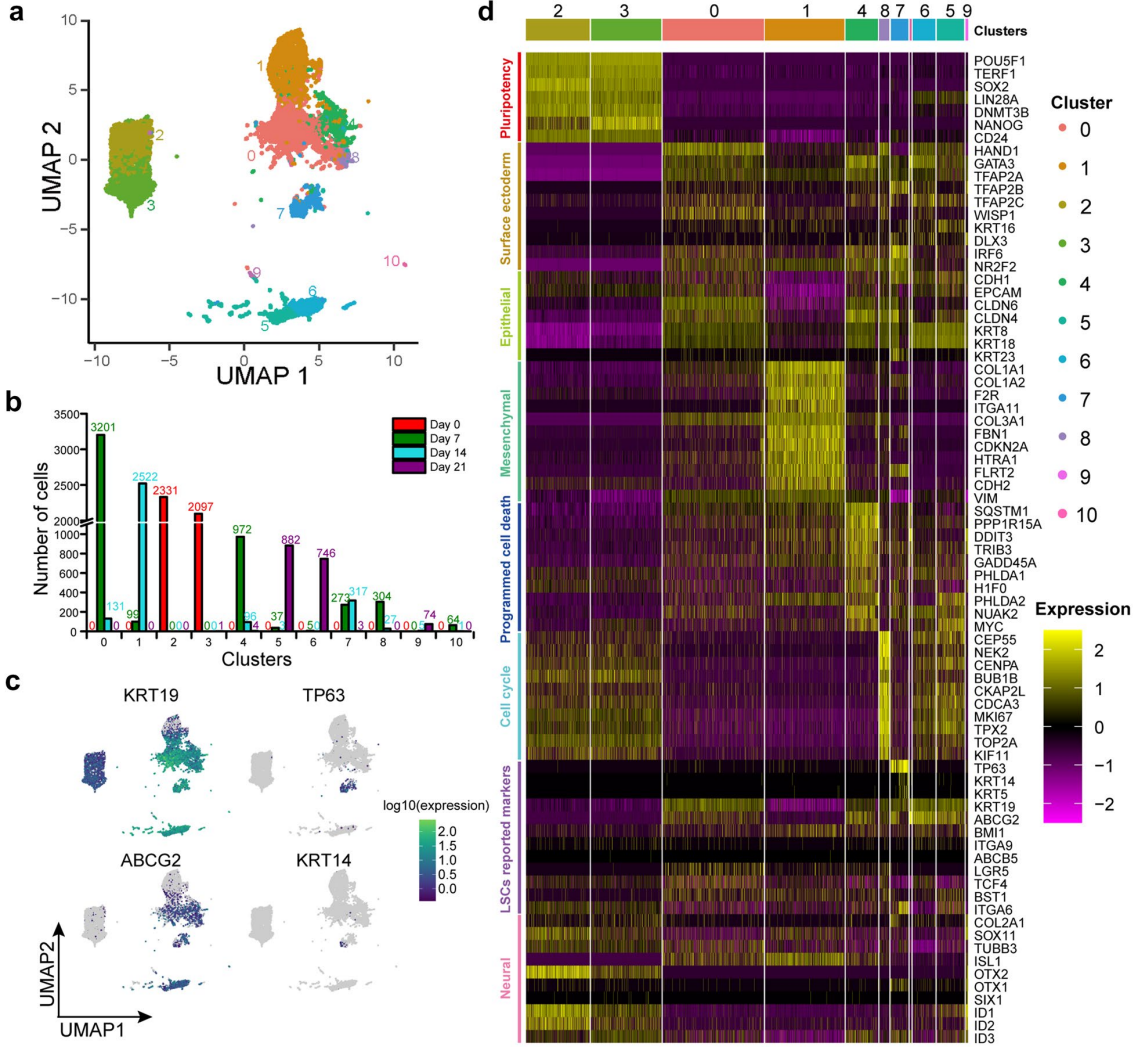
Time-course single-cell RNA sequencing profiling reveals heterogeneity of hESCs-derived LSCs. **a** UMAP visualizing the results of clustering for cells sequenced at the four times. **b** Barplot showing number of cells for the four time points in each cluster. **c** Feature plots visualizing expression of the four key LSC marker genes during differentiation of hESCs-derived LSCs. **d** Heatmap representing differentially expressed genes related to specific biological processes or cell types among the clusters

To investigate transcriptional changes during hESCs to LSCs differentiation, we integrated data from the four time points for dimension reduction and visualization. Results showed that the cells were grouped into 11 clusters (Fig. 2a). Among the clusters, cluster 2 and 3 were from Day 0 (Fig. 2b). Not surprisingly, these cells exhibited highest expression level of pluripotent genes POU5F1, SOX2, NANOG, and DNMT3B (Fig. 2d). In contrast, expression of surface ectodermal genes, such as TFAP2A, TFAP2B, TFAP2C, HAND1, GATA3, IFR6, WISP1, and NR2F2, were upregulated throughout differentiation (Fig. 2d). Unexpectedly, epithelial genes such as CDH1, EPCAM, KRT8, and KRT18, were lowly expressed in cluster 1, while mesenchymal genes such as CDH2, COL1A1, COL1A2, and FBN1 were highly expressed, indicating that cluster 1 were mesenchymal cells. In addition, neural genes, such as COL2A1, SOX11, OTX1 and SIX1, were upregulated in cluster 9 (Fig. 2d). These results demonstrated that during this LSCs induction process, hESCs gave rise to cells with none epithelial characteristics.

Notably, differential gene expression (DGE) analysis showed that genes related to cell cycle and programmed cell death were highly expressed in cluster 8 and cluster 4, respectively (Fig. 2d). In cluster 8, expression of genes related to cell cycle such as TOP2A, MKI67, TPX2, BUB1B, and CEP55 were significantly upregulated, while SQSTM1, DDIT3, PPP1R15A, H1F0, and TRIB3 etc., which are involved in programmed cell death, showed higher expression in cluster 4 (Fig. 2d). To avoid the potential bias from cell cycle effects, we assigned cell cycle phase to each cell. Then, we only extract cells in G2M phase to compare the expression of cycle related genes. Results demonstrated that cycle related genes, such as TOP2A, MKI67, TPX2, BUB1B, and CEP55 etc., were highly expressed in cluster 8 as well (Additional file 1: Fig. S2d, e). These results demonstrated that there were no obvious cell cycle effects on data dimension reduction and cluster 8 were indeed rapidly proliferating cells.

Next, we investigated expression of several putative LSC-associated markers (e.g. KRT19, ABCG2, VIM, ITGA9, TP63, KRT14, KRT15, KRT5) and differentiation-associated markers (e.g. KRT3 and KRT12) [14, 33] during hESC-derived LSCs differentiation. Results showed that differentiation-associated markers KRT3 and KRT12 were not detected in all clusters. Interestingly, putative LSC-associated markers TP63 and KRT14 were highly expressed in cluster 7 while KRT19 and ABCG2 were upregulated in all the clusters except cluster 1 (Fig. 2c, d). Taken together, these results indicated that cells in cluster 0, cluster 5, cluster 6, cluster 7, cluster 8, and cluster 10 could be progenitors of LSCs, LSCs or their progeny in the different stages of development.

### Pseudotime analysis revealed unique hESC-derived LSCs developmental trajectory

**Fig. 3:**
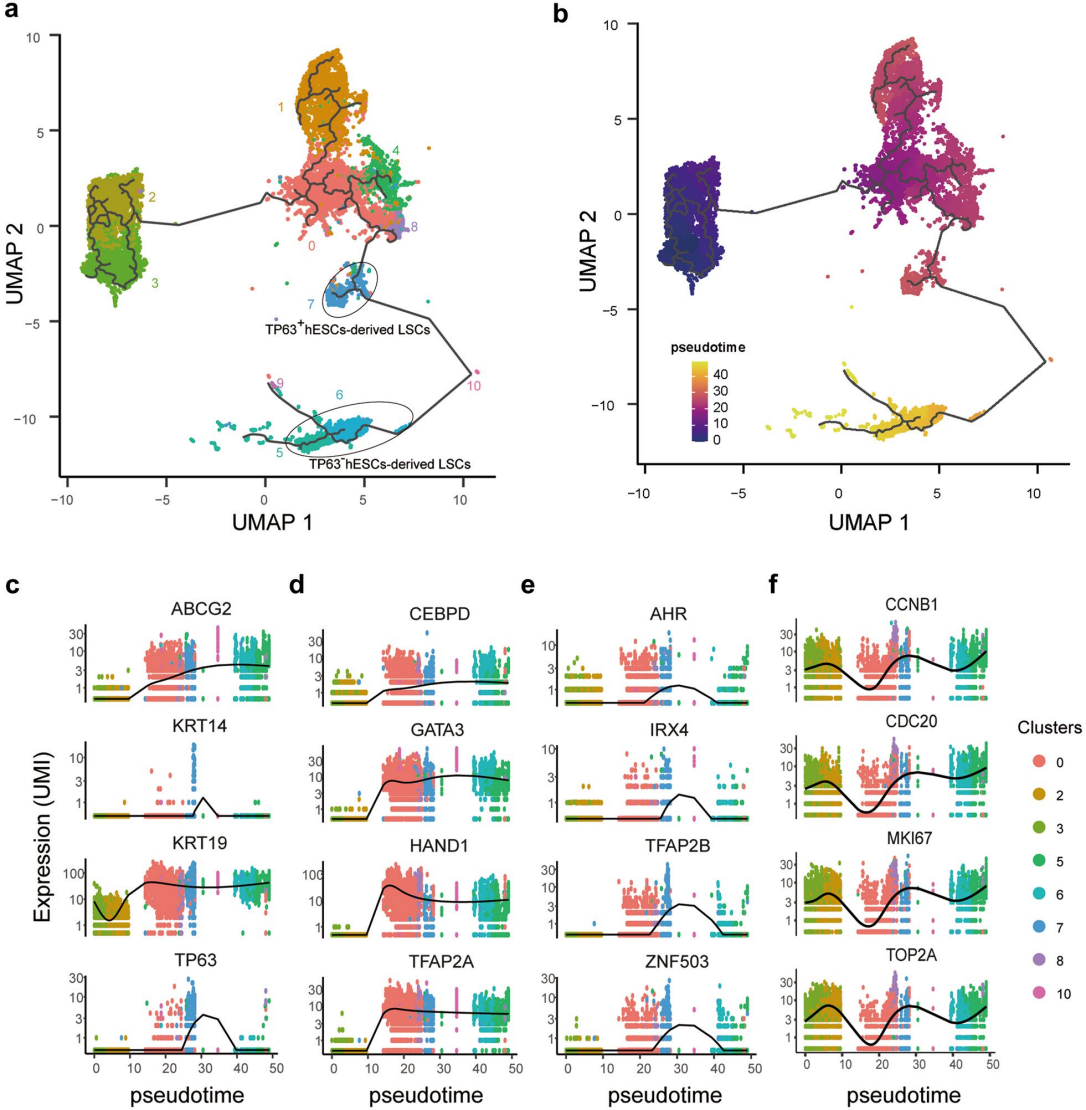
Pseudotime analysis characterizes expression changes throughout hESCs-derived LSCs differentiation. **a, b** UMAP visualizing developmental trajectories of cells for each cluster (**a**) and pseudotime assigned to each cell (**b**). **c** Spline plots showing expression changes of expression for the four candidate LSC marker genes, ABCG2, KRT14, KRT19, and TP63, over hESC-derived LSCs differentiation pseudotime. **d, e** Spline plots representing changes of expression for TFs upregulated upon differentiation and continually highly expressed (**d**), and upregulated in certain period (**e**) over hESC-derived LSCs pseudotime. **f** Spline plotts representing changes of expression for cell cycle related genes over hESC-derived LSCs pseudotime

To investigate hESC-derived LSCs developmental trajectory, we performed pseudotime analysis to study the path and progress of individual cells undergoing hESCs-derived LSCs differentiation [34]. The resultant trajectory indicated that a trifurcation point in cluster 0 could lead to cells fate commitment toward cluster 1 (Branch 1), cluster 4 (Branch 2), and cluster 8 that further differentiate to cells in cluster 7, 10, 5 and 6 (Branch 3) (Fig. 3a, b).

In Branch 1, CDH1 (E-cadherin) and CDH2 (N-cadherin), two well-known cadherins, were differently expressed between cells in cluster 0 and cluster 1 (Additional file 1: Fig. S3b, c). Specifically, CDH1 was upregulated in cluster 0 while CDH2 was expressed significantly higher in cluster 1. The loss of epithelial surface marker CDH1 and the acquisition of mesenchymal marker CDH2 is considered as the hallmark of epithelial-mesenchymal transition (EMT), which play pivotal role in developmental regulation, such as neural crest formation [35]. Additionally, upregulated genes in cluster 1 were significantly overrepresented in nervous system development (Additional file 1: Fig. S3d), indicating the possible generation of neural crest like cells during the hESCs to LSCs differentiation process. In Branch 2, cells were undergoing programmed cells death (apoptosis) as mentioned in the section above (Additional file 1: Fig. S3d). Apoptosis is a positive regulator of stem cells populations, it plays fundamental roles in development and tissue homeostasis [18, 36].

Branch 3 identified the main hESC-derived LSCs developmental trajectory (Fig. 3a). Epithelium development and epithelial cell proliferation related genes were upregulated in the Branch 3 differentiation process (Additional file 1: Fig. S3d). Increased expression of candidate LSC markers KRT19, ABCG2, KRT14, and TP63 were seen in cluster 5, cluster 6, and cluster 7 (Fig. 2c). Pseudotime analysis further demonstrated that these candidate markers exhibiting different trajectory patterns in Branch 3 (Fig. 3c). In addition, some transcription factors (TFs), such as CEBPD, GATA3, HAND1, and TFAP2A, were upregulated upon differentiation and stably expressed at high level (Fig. 3d), while some TFs, such as AHR, IRX4, TFAP2B, and ZNF530, only upregulated in a certain period of time like TP63 (Fig. 3c, e), indicating their distinct roles in hESC-derived LSCs development. According to expression of TP63, cells in cluster 7 could be assigned as TP63^+^ hESC-deirved LSCs while cells in cluster 5 and cluster 6 were TP63^−^ hESC-deirved LSCs (Fig. 3a). Interestingly, cell cycle related genes, such as CCNB1, CDC20, MKI67, and TOP2A, showed regular oscillations patterns across hESC-derived LSCs developmental pseudotime and regulate the cell proliferation and differentiation (Fig. 3f).

### Transcriptional difference of subpopulations in hESCs-derived LSCs

According to the expression comparisons, cluster 7 expressed most reported candidate LSC markers, including TP63 [37], KRT14 [38], KRT15 [39], ITGA6 [40] etc. (Additional file 1: Fig. S4a and Additional file 2). To investigate expression differences among subpopulations in hESCs-derived LSCs, we further focused on two-two comparisons among cluster 5, cluster 6, and cluster 7 (Additional file 1: Fig. S4b). Differential expression analysis demonstrated that upregulated genes in cluster 5 and cluster 6 showed significant enrichment in cell cycle process. In addition, genes involved in cell migration regulation were highly expressed in cluster 5 compared to cluster 6, including cadherin genes CDH5 and CDH13, integrin genes ITGA2, ITGA6, ITGA3, ITGB6, ITGB1, ITGA5 and ITGAV, collagen genes COL4A1, COL4A2, COL1A2 and COL3A1, and transcription factors SOX9, MYC, STAT3 etc. (Additional file 2). “X, Y, Z hypothesis” of corneal epithelial maintenance suggested that proliferation of basal cells (X) and migration of centripetal cells can replace lost cells from the ocular surface (Z) to support the corneal epithelial homeostasis [41]. Within the cornea, nuclear p63 (TP63) is expressed by the basal cells of the limbal epithelium, but not by the transit amplifying cells (TACs)s covering the corneal surface [37]. Therefore, these results suggested that cells in cluster 7 (TP63^+^ hESCs-derived LSCs) give rise to TACs (TP63^−^ hESCs-derived LSCs) in cluster 5 and cluster 6 (Fig. 3a), both of which are the progeny of LSCs exhibiting higher, but limited proliferative activity [37, 42].

**Fig. 4:**
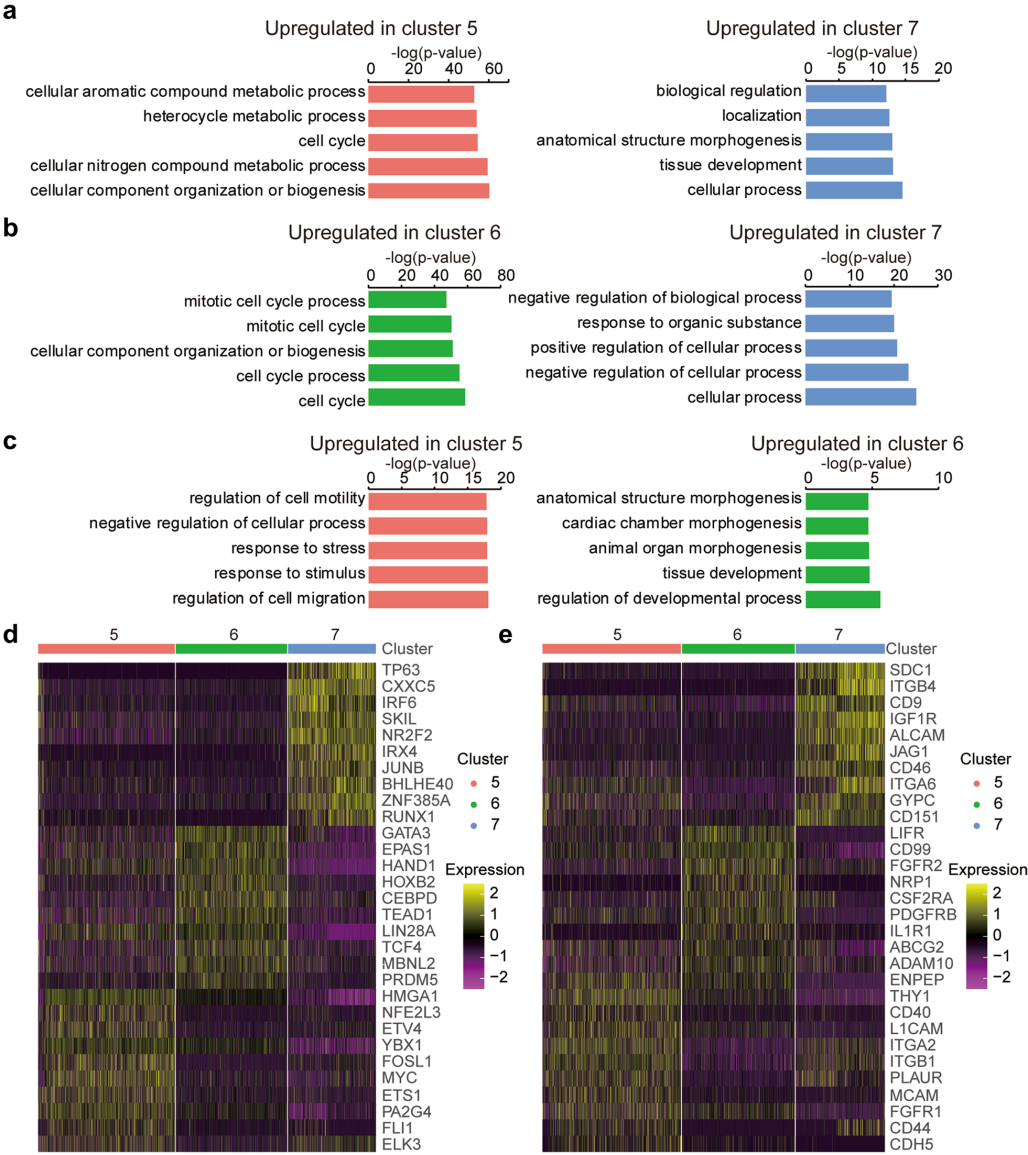
Transcriptional difference of subdipopulations in hESCs-derived LSCs. **a-c** Barplots showing GO biological process enrichment for upregulated genes compared between cluster 5 and cluster 7 (**a**), between cluster 6 and cluster 7 (**b**), and between cluster 5 and cluster 6 (**c**). Five terms with lowest p-value were presented. **d** Heatmap representing differentially expressed TFs among cluster 5, cluster 6, and cluster 7. **e** Heatmap representing differentially expressed top CD genes among cluster 5, cluster 6, and cluster 7. Wilcoxon Rank Sum test were performed for significant test and ten genes with lowest p_val_adj were presented

To identify potential markers to distinguish these cells, we focused on transcription factors (TFs) and cluster of differentiation (CD) genes differentially expressed in cells from cluster 5, cluster 6, and cluster 7. Among the TFs, CXXC5, IRF6, SKIL, RUNX1 etc. as well as TP63 were upregulated in cluster 7. GATA3, EPAS1, HAND1, HOXB2, and CEBPD etc. were highly expressed in cluster 6, while NFE2L3, EVT4, YBX1, FOSL1, and MYC etc. were enriched in cluster 5 (Fig. 4d). As to the CD genes, SDC1, ITGB4, CD9, IGF1R, JAG1, CD46, CD151 etc. were highly expressed in cluster 7, while LIFR, CD99, FGFR2, ABCG2 etc. were upregulated in cluster 6, and ENPEP, THY1, CD40, CD44, CDH5 etc. exhibited highest expression in cluster 5 (Fig. 4e). All these candidate markers identified here would be valuable for future investigation and characterization of different cell types in human cornea.

## Discussion

In this study, we performed a time-course transcriptome profiling of hESC-derived LSCs to revealed their gene expression changes and developmental trajectory at the single-cell level. Previous studies have shown that *bona fide* LSCs have the potential to establish and maintain long-term corneal repair. The identity of human LSCs have been investigated, and several candidate LSCs markers have been identified, such as TP63 [37], KRT14 [38], ITGA6 [40], NTRK1 [43], ABCG2 [30, 31], KRT15 [39], ABCB5 [44]. Besides, the terminally differentiated markers KRT3 and KRT12 were absent in LSCs [14, 45]. However, according to our single cell expression profiling data, hESC-derived LSCs showed significantly cellular heterogeneity using current protocols in our laboratory. For example, TP63 expressed cells only accounted for 6.034% of cells at Day 7, 11.21% of cells at Day 14, and 2.46% at Day 21 (Fig. 1e). Although several studies have successfully established candidate LSCs derived from human ESCs or iPSCs [10, 15, 28, 46–51], these protocols of differentiation are still time-consuming, expensive, or inefficient [52]. In addition, the reproducibility of differentiation is still one of the major challenges for in vitro LSCs induction, which may be affected by many factors, such as different differentiation propensities of cell lines or clones, batch effects in materials, different markers for efficiency and potency testing, etc. [53]. In this study, LSCs were differentiated from human ESCs according to the improved protocols [15, 28], which were based on replicating signaling cues by small-molecule inhibitors and activators to promote ocular surface ectoderm development. This protocol is more compliant to good manufacturing practice standards for the future possible clinical applications. Although high differentiation efficiency was reported in the protocols we referred, our data indicated a complex developmental trajectory and the existence of heterogenic subpopulations which need to be further characterized. And the current hESCs-derived LSCs differentiation methods with different cell lines need to be optimized.

Until recently, the developmental origin of LSCs remained elusive [14], and LSCs could be developmental descendants of the surface ectoderm as well as the periocular mesenchyme. Our scRNA-seq data revealed that EMT program were activated in the cluster of cells with neural crest characteristics at early hESC-derived LSCs differentiation stage (Additional file 1: Fig. S3). During organogenesis, epithelial cells can give rise to mesenchymal cells through EMT while the reverse process, mesenchymal–epithelial transition (MET), can generate epithelial cells [54], suggesting LSCs could be differentiated from the periocular mesenchyme through MET. However, our pseudotime trajectory analysis showed that induced mesenchymal cells did not generate LSCs under current culture conditions, and whether the periocular mesenchyme could give rise to LSCs remain to be confirmed. Meanwhile, we found excessive cell detachment occurred in cells cultured in the medium beyond 20 days, indicating the medium used need to be improved for LSCs generation and maintenance. Nevertheless, our pseudotime analysis identified a hESC-derived LSCs developmental trajectory. According to the trajectory, cell cycle related genes, such as CCNB1, CDC20, MKI67, and TOP2A, showed variable expression across hESC-derived LSCs developmental pseudotime (Fig. 3f). During organogenesis, cell cycle modulation is important for cell fate determination [55].

For long term restoration of visual function caused by LSCD, LSCs based transplantation either through autologous or allogenic grafting of limbal tissue, or cultured and expanded limbal cells have already shown effectiveness in the treatment [8]. However, so far, only TP63 positive LSCs were reported to be associated with therapeutic success [29]. But TP63 could not be applied to sort pure population of LSCs, and isolation of pure LSCs is still the bottleneck concerning the clinical application of LSCs. Therefore, other molecular markers are needed for successful prospective enrichment of LSC cells capable of long-term corneal restoration [14]. Identification of specific biomarkers for isolating and characterizing LSCs is crucial for both understanding their basic biology and translating in clinical application [14, 19]. According to our scRNA-seq data, TP63 expressed LSCs showed relative quiescence compared to their progenies, and genes related to cell cycle were significantly upregulated in highly proliferative progenies (TACs), which are in line with previous reports that epithelial stem cells are relatively quiescent and give rise to TACs [56]. Besides reported markers—TP63 and ITGA6, TFs such as CXXC5, IRF6, SKIL, NR2F2, IRX4 etc., and CD genes such as SDC1, CD9, IGF1R, ALCAM etc., were newly identified as potential markers that highly expressed in TP63 expressed hESC-derived LSCs (Fig. 4c, d). Thus, these data provided valuable sources for characterization of LSCs and optimization of hESC-derived LSCs differentiation protocols.

## Conclusions

In summary, we studied the time-course gene expression changes during hESC-derived LSCs differentiation in vitro at the single-cell level, and revealed significant transcriptional heterogeneity. Based on current differentiation protocol used in this study, expression heterogeneity of reported LSC markers were identified in subpopulations of differentiated cells. EMT has been shown to occur during differentiation process, which could possibly result in generation of untargeted cells. Pseudotime trajectory revealed transcriptional changes and signatures of commitment for LSCs and their progeny (TACs) that derived from pluripotent stem cells. Furthermore, some new potential markers for LSCs were identified, which are valuable for future investigation to elucidate identity and developmental origin of human LSCs.

## Methods

### Cell culture

The Ethics Committee of BGI-IRB approved this study. Human ESC lines H9 were cultured as previous description [57]. Briefly, cells were retrieved from liquid nitrogen tank and cultured in hESC medium (DMEM/F12 basic medium (Life Technologies), 20% knockout serum replacement (KSR, Life Technologies), 1 × L-glutamine (Life Technologies), 1 × MEM NEAA (Life Technologies), 0.1 mM 2-Mercaptoethanol (Life Technologies) and 50 ng/mL human FGF-2 (Life Technologies)) on mitomycin C (Sigma) treated murine embryonic fibroblasts (MEFs). To sustain undifferentiated states, cells were fed daily with fresh medium. For passaging, colonies were dispersed into small clumps with 1 mg/mL Collagenase IV (Life Technologies) for 20 min at 37℃, then plated onto Matrigel hESC-qualified Matrix (Corning)-coated dishes in mTeSR1 medium (Stemcell Technologies) at a ratio of 1:3 to 1:6. In the feeder-free medium, ReLeSR™ (Stemcell Technologies) were used for dissociation and passaging according to the manual.

### LSCs induction

LSCs were differentiated from human ESCs according to the published protocols with some changes [15, 28]. Briefly, when colonies reaching about 80–90% confluency, ReLeSR™ were used to digest cells into clumps. Then, these clumps were suspended in LSCs induction medium (DMEM/F12 basic medium, supplemented with 20% KSR, 1 × L-glutamine, 1 × MEM NEAA, 0.1 mM 2-Mercaptoethanol) adding 10 μM Y-27632 (Sigma) at 37℃ to induce embryoid body (EB) formation overnight. For LSCs differentiation, EBs were cultured in LSCs induction medium supplemented with 10 μM SB-505124 and 50 ng/ml FGF-2 for 1 day. Then, medium changed with LSCs induction medium supplemented 25 ng/ml bone morphogenetic protein 4 (BMP4) (R&D) for 2 days. Thereafter, the induced cultures were seeded onto plates coated with 0.75 μg/cm2 LN521 (BIOLAMINA) and 5 μg/cm2 col IV (Sigma) in a defined and serum-free medium CnT-30 (CELLNTEC). For next days before collection for scRNA-seq, the cells were maintained in CnT-30 and change the medium every 3 days.

### scRNA-seq library construction and sequencing

scRNA-seq experiments were performed by Chromium Single Cell 5′ Library & Gel Bead Kit (10 × Genomics), according to the manufacturer’s protocol. Briefly, cells were digested with TrypLE™ Select (ThermoFisher Scientific) and single cell suspension were harvested, washed with PBS twice, and filtered by 40 μm cell strainers (BD Falcon) before Gel Bead-In Emulsions (GEMs) generation and barcoding. Single-cell RNA-seq libraries were obtained following the 10 × Genomics recommended protocol, using the reagents included in the kit. Libraries were sequenced on the BGISEQ-500 (BGI) instrument [58] using 26 cycles (cell barcode and UMI [59]) for read1 and 108 cycles (sample index and transcript 5′ end) for read2.

### scRNA-seq Analysis

#### Quality control

The scRNA-seq data were processed using cellranger-3.0.2 for each sample with default parameters mapping to the human GRCh38 genome expect the number of recovered cells (–expect-cells option) was set to 8 000.

For each library, we filtered outlier cells using the median absolute deviation from the median total library size (logarithmic scale), total gene numbers (logarithmic scale), as well as mitochondrial percentage, as implemented in scran, using a cutoff of 3 (isOutlier, nmads = 3) [60]. For filtering lowly or none expressed genes, genes expressed across all the cells detected in less than 10 cells were removed, and totally 22 501 genes were kept for downstream analysis. Then, clean gene-cell UMI count matrix was loaded as Seurat object using R package Seurat 3.0 [61] or cds object using R package monocle 3 [62] to manage our dataset for the further analysis with default parameters otherwise will be mentioned in detail.

#### Cell cycle phase assignment

To assign cell cycle phase, cell cycle scores (i.e., G2/M scores and S scores) and phases (i.e. G1, G2/M, and S) for each cell on the basis of scores using function CellCycleScoring from R package Seurat based on the expression levels of a panel of phase-specific marker genes [63].

#### Normalization and dimension reduction

The quality controlled dataset was then analyzed using the Seurat v.3.0 pipeline with NormalizeData function to normalize our data, FindVariableFeatures funtion to assign top 2000 highly variably expressed genes, ScaleData function of argument vars.to.regress to remove confounding sources of variation (variables to be regressed out including mitochondrial mapping percentage, number of UMI). Following normalization and scaling, RunPCA function were performed to capture principal components using the top 2000 highly variably expressed genes. UMAP was applied to visualize and explore data in two-dimensional coordinates, generated by RunUMAP function in Seurat.

#### Cell cluster

For cell clustering, a graph-based clustering approach [61] were used to cluster the cells into candidate subpopulations. The first 50 PCs in the data were applied to construct an SNN matrix using the FindNeighbors function in Seurat v3 with k.param set to 20. We then identified clusters using the FindClusters command with the resolution parameter set to 0.5.

#### Differential expression analysis

To find differential expressed genes (DEGs), Wilcoxon Rank Sum test were performed for significant test using Seurat function FindAllMarkers for each cluster compared to all remaining cells and FindMarkers for distinguishing each other. Genes with average natural log fold change more than 0.25 and FDR less than 0.01 were assigned as DEGs.

#### Pseudotime trajectories analysis

For pseudotime trajectories analysis, the quality control dataset with cell clustering information were analyzed using the monocle3 (http://cole-trapnell-lab.github.io/ monocle3/) pipeline. The new_cell_data_set function in the package was used to create cds object, and preprocess_cds function was applied for data normalization and principal component analysis with num_dim setting to 50. Then, reduce_dimension, cluster_cells, and learn_graph functions were used for data reduction, cell clustering, and pseudotime trajectories construction, respectively. UMAP was applied to visualize and explore data in two-dimensional coordinates using plot_cells function.

## Supplementary Information

**Additional file 1: Figure S1.** Overview of the experimental procedure and data quality. **Figure S2.** Cell cycle phase assigned for cells throughout hESCs-derived LSCs differentiation. **Figure S3.** Pseudotime analysis characterizes expression changes throughout hESCs-derived LSCs differentiation. **Figure S4.** Transcriptional difference of subpopulations in hESCs-derived LSCs.

**Additional file 2: Table S1.** List of DEGs between cluster 8 and cluster 0. **Table S2.** List of DEGs between cluster 7 and cluster 0. **Table S3.** List of DEGs between cluster 6 and cluster 0. **Table S4.** List of DEGs between cluster 5 and cluster 0. **Table S5.** List of DEGs between cluster 7 and cluster 6. **Table S6.** List of DEGs between cluster 6 and cluster 8. **Table S7**. List of DEGs between cluster 5 and cluster 8. **Table S8.** List of DEGs between cluster 6 and cluster 7. **Table S9.** List of DEGs between cluster 5 and cluster 7. **Table S10.** List of DEGs between cluster 6 and cluster 5. 

## Data Availability

The data that support the findings of this study have been deposited into CNGB Sequence Archive (CNSA) [64] of China National GeneBank DataBase (CNGBdb)[65] with accession number CNP0001218.

## References

[1] Davanger M, Evensen A (1971). Role of the pericorneal papillary structure in renewal of corneal epithelium. Nature.

[2] Busin M, Breda C, Bertolin M, Bovone C, Ponzin D, Ferrari S (2016). Corneal Epithelial Stem Cells Repopulate the Donor Area within 1 Year from Limbus Removal for Limbal Autograft. Ophthalmology.

[3] Kawakita T, Higa K, Shimmura S, Tomita M, Tsubota K, Shimazaki J (2011). Fate of corneal epithelial cells separated from limbus in vivo. Invest Ophthalmol Vis Sci.

[4] Ebrahimi M, Taghi-Abadi E, Baharvand H (2009). Limbal stem cells in review. J Ophthalmic Vis Res.

[5] Barut Selver O, Yagci A, Egrilmez S, Gurdal M, Palamar M, Cavusoglu T (2017). Limbal Stem Cell Deficiency and Treatment with Stem Cell Transplantation. Turk J Ophthalmol.

[6] Le Q, Xu J, Deng SX (2018). The diagnosis of limbal stem cell deficiency. Ocul Surf.

[7] Kim KH, Mian SI (2017). Diagnosis of corneal limbal stem cell deficiency. Curr Opin Ophthalmol.

[8] Atallah MR, Palioura S, Perez VL, Amescua G (2016). Limbal stem cell transplantation: current perspectives. Clinical Ophthalmol.

[9] Kamarudin TA, Bojic S, Collin J, Yu M, Alharthi S, Buck H (2018). Differences in the activity of endogenous bone morphogenetic protein signaling impact on the ability of induced pluripotent stem cells to differentiate to corneal epithelial-like cells. Stem Cells.

[10] Hayashi R, Ishikawa Y, Sasamoto Y, Katori R, Nomura N, Ichikawa T (2016). Co-ordinated ocular development from human iPS cells and recovery of corneal function. Nature.

[11] Hanson C, Hardarson T, Ellerstrom C, Nordberg M, Caisander G, Rao M (2013). Transplantation of human embryonic stem cells onto a partially wounded human cornea in vitro. Acta Ophthalmol.

[12] Ahmad S, Stewart R, Yung S, Kolli S, Armstrong L, Stojkovic M (2007). Differentiation of human embryonic stem cells into corneal epithelial-like cells by in vitro replication of the corneal epithelial stem cell niche. Stem Cells.

[13] Chakrabarty K, Shetty R, Ghosh A (2018). Corneal cell therapy: with iPSCs, it is no more a far-sight. Stem Cell Res Ther.

[14] Gonzalez G, Sasamoto Y, Ksander BR, Frank MH, Frank NY. Limbal stem cells: identity, developmental origin, and therapeutic potential. Wiley Interdiscip Rev Dev Biol. 2018;7(2). 10.1002/wdev.303 PMC5814333 29105366

[15] Hongisto H, Ilmarinen T, Vattulainen M, Mikhailova A, Skottman H (2017). Xeno- and feeder-free differentiation of human pluripotent stem cells to two distinct ocular epithelial cell types using simple modifications of one method. Stem Cell Res Therapy.

[16] Tchieu J, Zimmer B, Fattahi F, Amin S, Zeltner N, Chen S (2017). A Modular Platform for Differentiation of Human PSCs into All Major Ectodermal Lineages. Cell Stem Cell.

[17] Wolosin JM, Budak MT, Akinci MA (2004). Ocular surface epithelial and stem cell development. Int J Dev Biol.

[18] Kaplan N, Wang J, Wray B, Patel P, Yang W, Peng H (2019). Single-Cell RNA transcriptome helps define the limbal/corneal epithelial stem/early transit amplifying cells and how autophagy affects this population. Invest Ophthalmol Vis Sci.

[19] Sonam S, Srnak JA, Perry KJ, Henry JJ. Molecular markers for corneal epithelial cells in larval vs. adult Xenopus frogs. Experimental Eye Research. 2019;184:107–25. 10.1016/j.exer.2019.04.010 PMC6697113 30981716

[20] Rossant J (2015). Mouse and human blastocyst-derived stem cells: vive les differences. Development.

[21] Chakrabarty K, Shetty R, Ghosh A (2018). Corneal cell therapy: with iPSCs, it is no more a far-sight. Stem Cell Res Therapy.

[22] Pattison JM, Melo SP, Piekos SN, Torkelson JL, Bashkirova E, Mumbach MR (2018). Retinoic acid and BMP4 cooperate with p63 to alter chromatin dynamics during surface epithelial commitment. Nat Genet.

[23] Gurtner GC, Hwang B, Lee JH, Bang D (2018). Single-cell RNA sequencing technologies and bioinformatics pipelines. Stem Cells.

[24] Tang F, Barbacioru C, Wang Y, Nordman E, Lee C, Xu N (2009). mRNA-Seq whole-transcriptome analysis of a single cell. Nat Methods.

[25] Su X, Shi Y, Zou X, Lu ZN, Xie G, Yang JYH (2017). Single-cell RNA-Seq analysis reveals dynamic trajectories during mouse liver development. BMC Genomics.

[26] Clark BS, Stein-O'Brien GL, Shiau F, Cannon GH, Davis-Marcisak E, Sherman T (2019). Single-Cell RNA-Seq Analysis of Retinal Development Identifies NFI Factors as Regulating Mitotic Exit and Late-Born Cell Specification. Neuron.

[27] Hu Y, Wang X, Hu B, Mao Y, Chen Y, Yan L (2019). Dissecting the transcriptome landscape of the human fetal neural retina and retinal pigment epithelium by single-cell RNA-seq analysis. PLoS Biol.

[28] Mikhailova A, Ilmarinen T, Uusitalo H, Skottman H (2014). Small-molecule induction promotes corneal epithelial cell differentiation from human induced pluripotent stem cells. Stem Cell Reports.

[29] Rama P, Matuska S, Paganoni G, Spinelli A, De Luca M, Pellegrini G (2010). Limbal stem-cell therapy and long-term corneal regeneration. N Engl J Med.

[30] de Paiva CS, Chen Z, Corrales RM, Pflugfelder SC, Li DQ (2005). ABCG2 transporter identifies a population of clonogenic human limbal epithelial cells. Stem Cells.

[31] Budak MT, Alpdogan OS, Zhou M, Lavker RM, Akinci MA, Wolosin JM (2005). Ocular surface epithelia contain ABCG2-dependent side population cells exhibiting features associated with stem cells. J Cell Sci.

[32] Vattulainen M, Ilmarinen T, Koivusalo L, Viiri K, Hongisto H, Skottman H (2019). Modulation of Wnt/BMP pathways during corneal differentiation of hPSC maintains ABCG2-positive LSC population that demonstrates increased regenerative potential. Stem Cell Res Therapy.

[33] Schlotzer-Schrehardt U, Kruse FE (2005). Identification and characterization of limbal stem cells. Exp Eye Res.

[34] Trapnell C, Cacchiarelli D, Grimsby J, Pokharel P, Li S, Morse M (2014). The dynamics and regulators of cell fate decisions are revealed by pseudotemporal ordering of single cells. Nat Biotechnol.

[35] Kim DH, Xing T, Yang Z, Dudek R, Lu Q, Chen YH. Epithelial Mesenchymal Transition in Embryonic Development, Tissue Repair and Cancer: A Comprehensive Overview. J Clin Med. 2017;7(1). 10.3390/jcm7010001 PMC5791009 29271928

[36] Fuchs Y, Steller H (2011). Programmed cell death in animal development and disease. Cell.

[37] Pellegrini G, Dellambra E, Golisano O, Martinelli E, Fantozzi I, Bondanza S (2001). p63 identifies keratinocyte stem cells. Proc Natl Acad Sci USA.

[38] Kurpakus MA, Maniaci MT, Esco M (1994). Expression of keratins K12, K4 and K14 during development of ocular surface epithelium. Curr Eye Res.

[39] Yoshida S, Shimmura S, Kawakita T, Miyashita H, Den S, Shimazaki J (2006). Cytokeratin 15 can be used to identify the limbal phenotype in normal and diseased ocular surfaces. Invest Ophthalmol Vis Sci.

[40] Hayashi R, Yamato M, Saito T, Oshima T, Okano T, Tano Y (2008). Enrichment of corneal epithelial stem/progenitor cells using cell surface markers, integrin alpha6 and CD71. Biochem Biophys Res Commun.

[41] Thoft RA, Friend J (1983). The X, Y, Z hypothesis of corneal epithelial maintenance. Invest Ophthalmol Vis Sci.

[42] Beebe DC, Masters BR (1996). Cell lineage and the differentiation of corneal epithelial cells. Invest Ophthalmol Vis Sci.

[43] Qi H, Li DQ, Shine HD, Chen Z, Yoon KC, Jones DB (2008). Nerve growth factor and its receptor TrkA serve as potential markers for human corneal epithelial progenitor cells. Exp Eye Res.

[44] Ksander BR, Kolovou PE, Wilson BJ, Saab KR, Guo Q, Ma J (2014). ABCB5 is a limbal stem cell gene required for corneal development and repair. Nature.

[45] Schermer A, Galvin S, Sun TT (1986). Differentiation-related expression of a major 64K corneal keratin in vivo and in culture suggests limbal location of corneal epithelial stem cells. J Cell Biol.

[46] Aberdam E, Petit I, Sangari L, Aberdam D (2017). Induced pluripotent stem cell-derived limbal epithelial cells (LiPSC) as a cellular alternative for in vitro ocular toxicity testing. PLoS ONE.

[47] Zhang C, Du L, Pang K, Wu X (2017). Differentiation of human embryonic stem cells into corneal epithelial progenitor cells under defined conditions. PLoS ONE.

[48] Brzeszczynska J, Samuel K, Greenhough S, Ramaesh K, Dhillon B, Hay DC (2014). Differentiation and molecular profiling of human embryonic stem cell-derived corneal epithelial cells. Int J Mol Med.

[49] Sareen D, Saghizadeh M, Ornelas L, Winkler MA, Narwani K, Sahabian A (2014). Differentiation of human limbal-derived induced pluripotent stem cells into limbal-like epithelium. Stem Cells Transl Med.

[50] Zhu J, Zhang K, Sun Y, Gao X, Li Y, Chen Z (2013). Reconstruction of functional ocular surface by acellular porcine cornea matrix scaffold and limbal stem cells derived from human embryonic stem cells. Tissue Eng Part A.

[51] Hayashi R, Ishikawa Y, Ito M, Kageyama T, Takashiba K, Fujioka T (2012). Generation of corneal epithelial cells from induced pluripotent stem cells derived from human dermal fibroblast and corneal limbal epithelium. PLoS ONE.

[52] Zhu J, Slevin M, Guo BQ, Zhu SR (2018). Induced pluripotent stem cells as a potential therapeutic source for corneal epithelial stem cells. Int J Ophthalmol.

[53] Hayashi R, Ishikawa Y, Katori R, Sasamoto Y, Taniwaki Y, Takayanagi H (2017). Coordinated generation of multiple ocular-like cell lineages and fabrication of functional corneal epithelial cell sheets from human iPS cells. Nat Protoc.

[54] Pei D, Shu X, Gassama-Diagne A, Thiery JP (2019). Mesenchymal-epithelial transition in development and reprogramming. Nat Cell Biol.

[55] Budirahardja Y, Gonczy P (2009). Coupling the cell cycle to development. Development.

[56] Lavker RM, Sun TT (2003). Epithelial stem cells: the eye provides a vision. Eye (Lond).

[57] Sun C, Zhang J, Zheng D, Wang J, Yang H, Zhang X (2018). Transcriptome variations among human embryonic stem cell lines are associated with their differentiation propensity. PLoS ONE.

[58] Natarajan KN, Miao Z, Jiang M, Huang X, Zhou H, Xie J (2019). Comparative analysis of sequencing technologies for single-cell transcriptomics. Genome Biol.

[59] Islam S, Zeisel A, Joost S, La Manno G, Zajac P, Kasper M (2014). Quantitative single-cell RNA-seq with unique molecular identifiers. Nat Methods.

[60] Lun AT, McCarthy DJ, Marioni JC (2016). A step-by-step workflow for low-level analysis of single-cell RNA-seq data with Bioconductor. F100Res..

[61] Macosko EZ, Basu A, Satija R, Nemesh J, Shekhar K, Goldman M (2015). Highly parallel genome-wide expression profiling of individual cells using nanoliter droplets. Cell.

[62] Cao J, Spielmann M, Qiu X, Huang X, Ibrahim DM, Hill AJ (2019). The single-cell transcriptional landscape of mammalian organogenesis. Nature.

[63] Nestorowa S, Hamey FK (2016). A single-cell resolution map of mouse hematopoietic stem and progenitor cell differentiation.

[64] Guo X, Chen F, Gao F, Li L, Liu K, You L, et al. CNSA: a data repository for archiving omics data. Database (Oxford). 2020;2020. 10.1093/database/baaa055 PMC7377928 32705130

[65] Chen FZ, You LJ, Yang F, Wang LN, Guo XQ, Gao F, et al. CNGBdb: China National GeneBank DataBase. Hereditas(Beijing). 2020;42(8):799–809. 10.16288/j.yczz.20-080 32952115

