# Supplementary material for: Time-course single-cell RNA sequencing reveals transcriptional dynamics and heterogeneity of limbal stem cells derived from human pluripotent stem cells
Source: Cell Biosci. 2021 Jan 23;11:24. doi: 10.1186/s13578-021-00541-4 (PMC7824938; doi:10.1186/s13578-021-00541-4)
Supplement: Supplementary file 1 — Additional file 1: Figure S1. Overview of the experimental procedure and data quality. Figure S2. Cell cycle phase assigned for cells throughout hESCs-derived LSCs differentiation. Figure S3. Pseudotime analysis characterizes expression changes throughout hESCs-derived LSCs differentiation. Figure S4. Transcriptional difference of subpopulations in hESCs-derived LSCs. [file 13578_2021_541_MOESM1_ESM.docx]

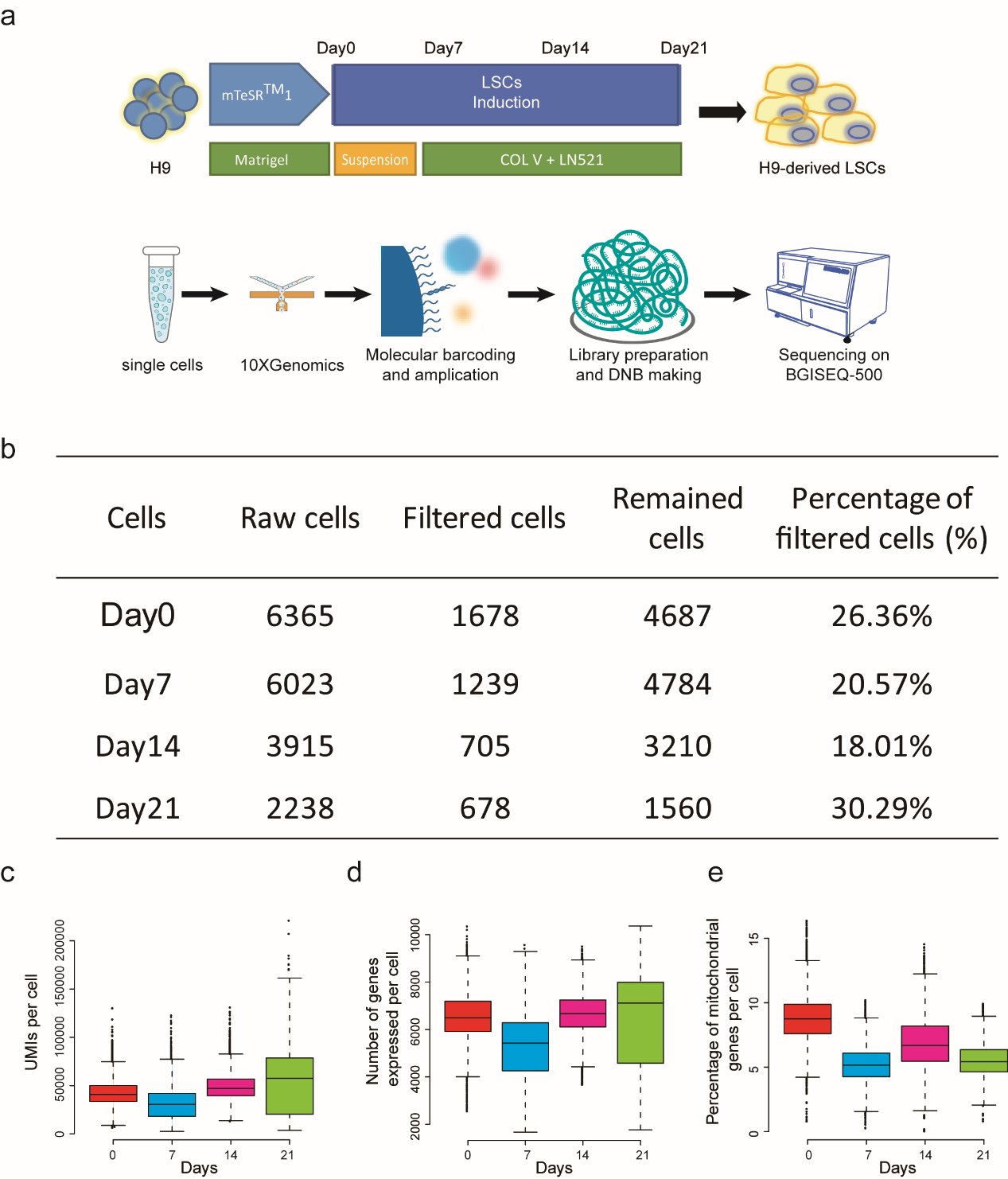


**Fig. S1** Overview of the experimental procedure and data quality. **a** Schematic representation of differentiation procedure, single cell RNA-seq library preparation, and sequencing for hESC-derived LSCs. **b** Number of cells sequenced and filtered for each time point. **c** Boxplot showing distribution on UMIs per cell for each time point. **d** Boxplot showing distribution on number of genes obtained per cell for each time point. **e** Boxplot showing distribution on percentage of mitochondrial genes per cell for each time point.


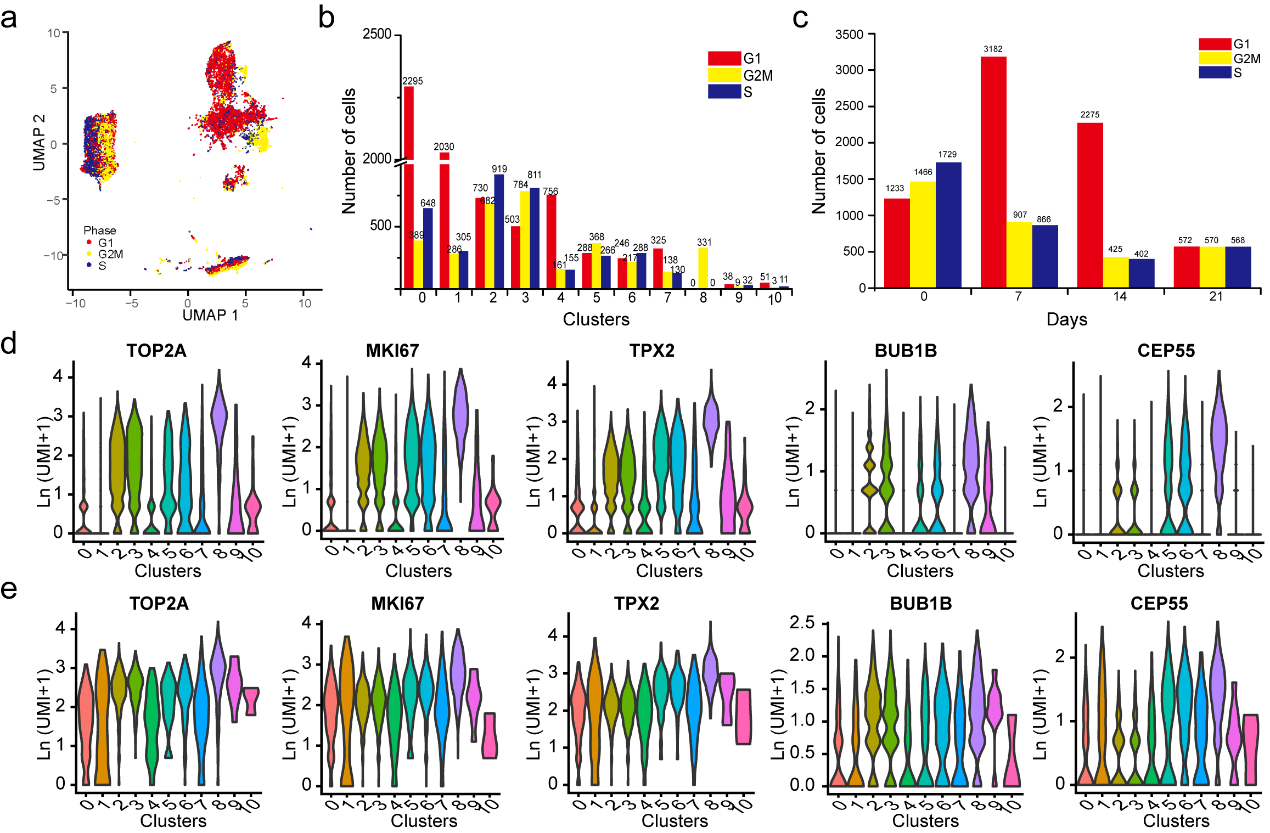


**Fig. S2** Cell cycle phase assigned for cells throughout hESCs-derived LSCs differentiation. **a** UMAP visualizing the results of cell cycle phases assigned for cells sequenced at the days 0, 7, 14, and 21. **b** Barplot showing number of cells assigned to the cell cycle phases for each cluster. **c** Barplot showing number of cells assigned to the cell cycle phases for each day. **d, e** Violin plots representing expression (ln (UMI+1)) of cell cycle related genes for cells only in G2M phase (**d**) and all cells (**e**) in each cluster.


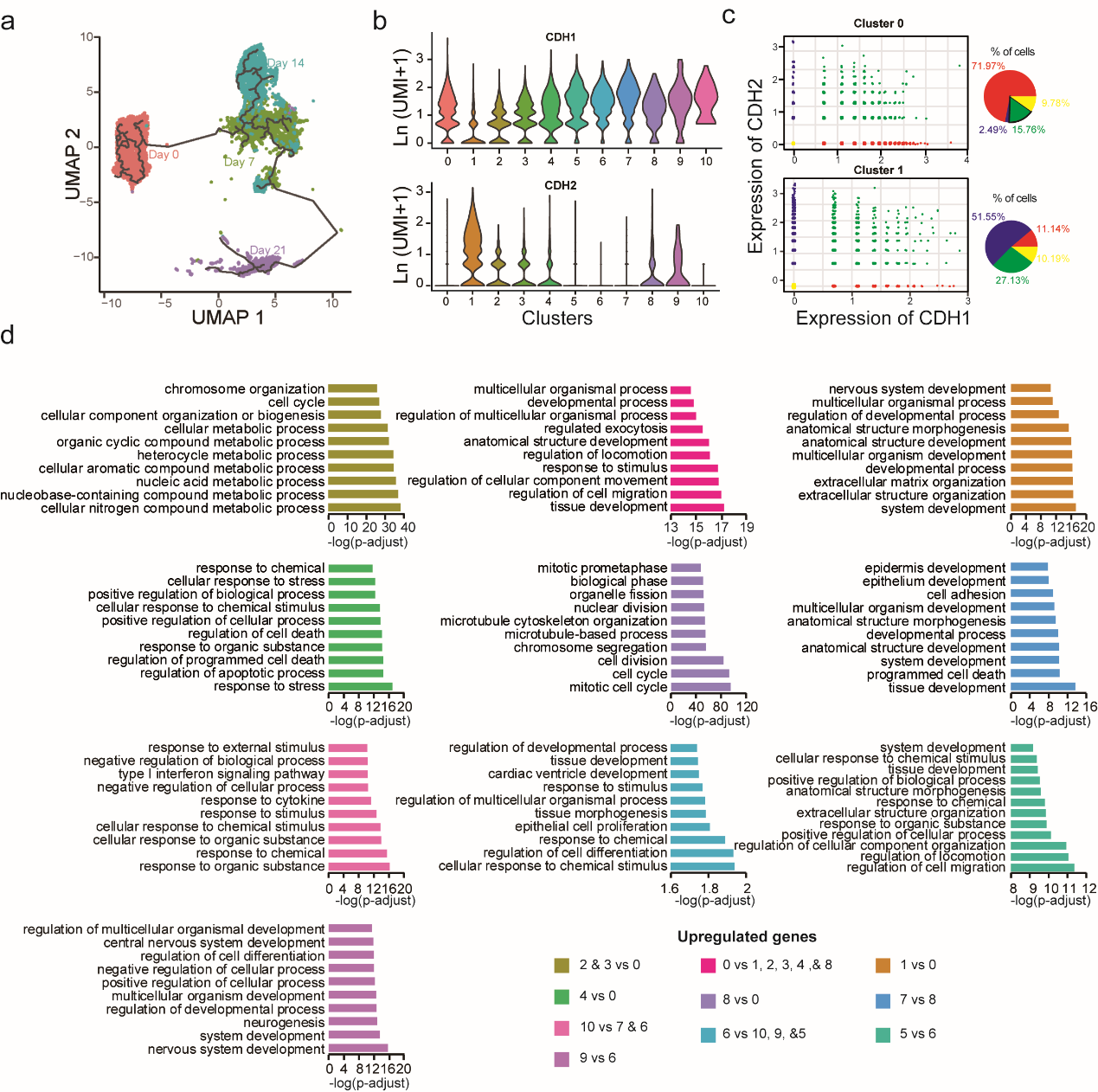


**Fig. S3** Pseudotime analysis characterizes expression changes throughout hESCs-derived LSCs differentiation. **a** UMAP visualizing developmental trajectory of cells in each day. **b** Violin plots showing distribution of expression (ln (UMI+1)) for CDH1 and CDH2 genes in each cluster. **c** Scatter plots showing expression (ln (UMI+1)) of CDH1 and CDH2 in each cell (left) and pie charts showing percentage of cells with different expression patterns of CDH1 and CDH2 genes for cluster 0 (up) and cluster 1 (down). **d** Barplots showing GO biological process enrichment for upregulated genes among adjacent clusters over the developmental trajectory. Only 10 terms with lowest p-adjust values were presented.


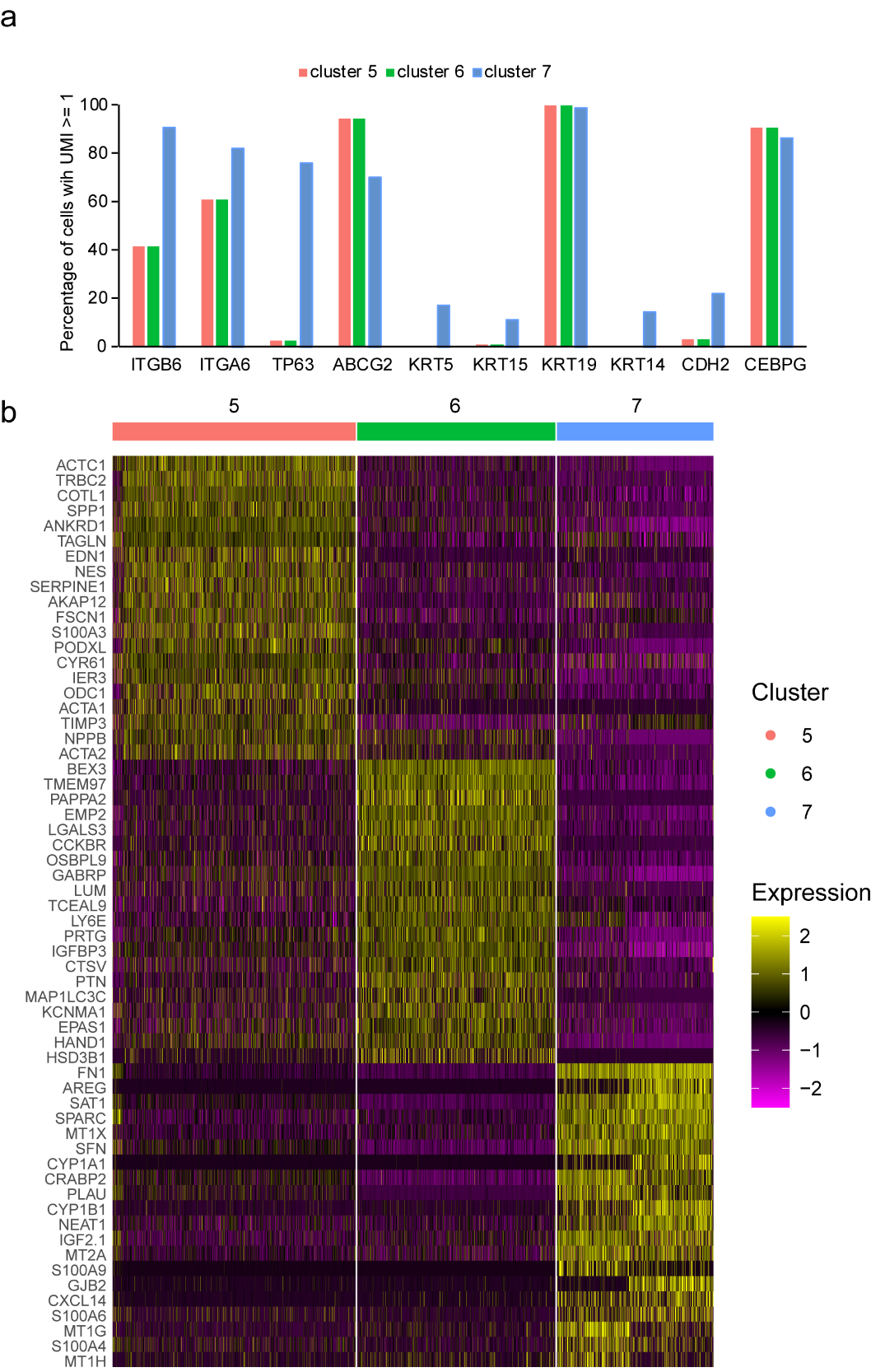


**Fig. S4** Transcriptional difference of subpopulations in hESCs-derived LSCs. **a** Barplot showing percentage of cells expressed some reported candidate LSCs markers (at least 1 UMI) in cluster 5, cluster 6, and cluster 7. **b** Heatmap representing differentially expressed genes among cluster 5, cluster 6, and cluster 7. Twenty genes with lowest p_val_adj were presented.
